# Novel Hemizygous *IL2RG* p.(Pro58Ser) Mutation Impairs IL-2 Receptor Complex Expression on Lymphocytes Causing X-Linked Combined Immunodeficiency

**DOI:** 10.1007/s10875-020-00745-2

**Published:** 2020-02-19

**Authors:** Elina A. Tuovinen, Juha Grönholm, Tiina Öhman, Sakari Pöysti, Raine Toivonen, Anna Kreutzman, Kaarina Heiskanen, Luca Trotta, Sanna Toiviainen-Salo, John M. Routes, James Verbsky, Satu Mustjoki, Janna Saarela, Juha Kere, Markku Varjosalo, Arno Hänninen, Mikko R. J. Seppänen

**Affiliations:** 1 Folkhälsan Research Center, Helsinki, Finland; 2 Translational Immunology Research Program, University of Helsinki, Helsinki, Finland; 3 Rare Diseases Center and Pediatric Research Center, New Children’s Hospital, University of Helsinki and HUS Helsinki University Hospital, Helsinki, Finland; 4 Systems Biology Research Group and Proteomics Unit, Institute of Biotechnology, HiLIFE, University of Helsinki, Helsinki, Finland; 5 Department of Clinical Microbiology and Immunology, Turku University Hospital and Institute of Biomedicine, University of Turku, Turku, Finland; 6 Hematology Research Unit Helsinki, Helsinki University Hospital Comprehensive Cancer Center, Helsinki, Finland; 7 Institute for Molecular Medicine Finland, HiLIFE, University of Helsinki, Helsinki, Finland; 8 Department of Pediatric Radiology, HUS Medical Imaging Center, Radiology, University of Helsinki and HUS Helsinki University Hospital, Helsinki, Finland; 9 Department of Pediatrics, Medical College of Wisconsin, Milwaukee, WI USA; 10 Department of Clinical Chemistry and Hematology, University of Helsinki, Helsinki, Finland; 11 Department of Medical Genetics, Helsinki Central University Hospital, Helsinki, Finland; 12 Centre for Molecular Medicine Norway, University of Oslo, Oslo, Norway; 13 Department of Biosciences and Nutrition, Karolinska Institutet, Stockholm, Sweden; 14 Stem Cells and Metabolism Research Program, University of Helsinki, Helsinki, Finland

**Keywords:** X-linked combined immunodeficiency diseases, severe combined immunodeficiency, atypical, interleukin receptor common gamma subunit, *IL2RG*, endoplasmic reticulum, Golgi apparatus

## Abstract

Hypomorphic *IL2RG* mutations may lead to milder phenotypes than X-SCID, named variably as atypical X-SCID or X-CID. We report an 11-year-old boy with a novel c. 172C>T;p.(Pro58Ser) mutation in *IL2RG*, presenting with atypical X-SCID phenotype. We also review the growing number of hypomorphic *IL2RG* mutations causing atypical X-SCID. We studied the patient’s clinical phenotype, B, T, NK, and dendritic cell phenotypes, IL2RG and CD25 cell surface expression, and IL-2 target gene expression, STAT tyrosine phosphorylation, PBMC proliferation, and blast formation in response to IL-2 stimulation, as well as protein-protein interactions of the mutated IL2RG by BioID proximity labeling. The patient suffered from recurrent upper and lower respiratory tract infections, bronchiectasis, and reactive arthritis. His total lymphocyte counts have remained normal despite skewed T and B cells subpopulations, with very low numbers of plasmacytoid dendritic cells. Surface expression of IL2RG was reduced on his lymphocytes. This led to impaired STAT tyrosine phosphorylation in response to IL-2 and IL-21, reduced expression of IL-2 target genes in patient CD4+ T cells, and reduced cell proliferation in response to IL-2 stimulation. BioID proximity labeling showed aberrant interactions between mutated IL2RG and ER/Golgi proteins causing mislocalization of the mutated IL2RG to the ER/Golgi interface. In conclusion, *IL2RG* p.(Pro58Ser) causes X-CID. Failure of IL2RG plasma membrane targeting may lead to atypical X-SCID. We further identified another carrier of this mutation from newborn SCID screening, lost to closer scrutiny.

**Electronic supplementary material:**

The online version of this article (10.1007/s10875-020-00745-2) contains supplementary material, which is available to authorized users.

## Introduction

X-linked severe combined immunodeficiency (X-SCID) accounts for approximately half of all the SCID cases and has an estimated incidence of 1:100000 male births [1]. It is caused by mutations in interleukin-2 receptor gamma chain (*IL2RG*) gene and presents with absent or profoundly diminished peripheral T and NK cells and functionally defective B cells [2, 3]. IL2RG is expressed by virtually all hematopoietic cells and is shared by the receptors for interleukins (IL) 2, 4, 7, 9, 15, and 21 [4]. In these receptors, it contributes to high-affinity ligand binding and coupling with the Janus kinase 3 (JAK3) with its cytoplasmic tail. Ligand binding induces phosphorylation-mediated activation of receptor-associated JAKs (1 and 3), further leading to JAK-mediated tyrosine phosphorylation, homodimerization, and nuclear translocation of signal transducers and activators of transcription (STATs) affecting target gene expression [5, 6].

The lack of normal IL-4, IL-7, IL-15, and IL-21 signaling explains the classical T^−^B^+^NK^−^ phenotype in X-SCID. IL-7 and IL-15 are required for T and NK cell development, respectively, and disturbed IL-4 and IL-21 signaling causes the intrinsic B cell defect [6–8]. IL-7 has a non-redundant role in T cell development; defective IL-7-induced signaling is the main reason for the T cell lymphopenia in X-SCID [9]. As a result, individuals with X-SCID lack adequate T cell function for survival [6, 10]. They present with early-onset respiratory tract infections, diarrhea, and failure to thrive. Without stem cell transplant or gene therapy, they usually die within the first year of life [2, 5].

Several cases with rare hemizygous *IL2RG* mutations and milder phenotypes, like X-linked combined immunodeficiency (CID) or common variable immunodeficiency (CVID), have been reported [2, 10–13]. Caused by hypomorphic mutations, genetic reversions in the early progenitor cells, or maternal T or NK cell engraftment, these atypical or “leaky” phenotypes may display preserved and/or partially functional T and NK cell subsets [3, 10, 12, 14–19]. Typical and atypical X-SCID have overlapping clinical features such as recurrent bacterial and viral infections, often caused by opportunistic pathogens. However, as the atypical X-SCID patients have greater amounts of residual T cell function, their clinical presentation is less severe and the onset usually later when compared to the classical X-SCID [10].

We report a boy with a novel c.172C>T;p.(Pro58Ser) mutation in *IL2RG*, with impaired expression on IL-2 receptor complex, and another boy with the same mutation caught in newborn SCID screening. Our index patient presented with an X-CID phenotype with normal numbers of T, B, and NK cells, he suffered from recurrent respiratory tract infections, bronchiectasis, and reactive arthritis.

## Methods

### Genetic Analyses

**Fig. 1:**
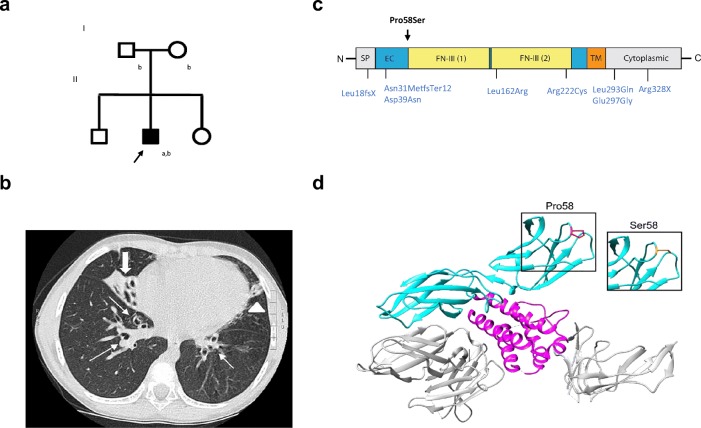
Patient family pedigree, high-resolution lung computed tomography and domain structure of IL2RG. **a** Family pedigree (^a^Whole exome sequencing; ^b^Targeted screening by Sanger sequencing). **b** High-resolution lung computed tomography at age 6 years demonstrated centrally located bronchiectasis in both lower lobes (thin arrows), a cluster of cystic bronchiectasis in the completely collapsed right middle lobe (thick arrow), a fibrotic strand with small caliper traction bronchiectasis in the left lingula (arrowhead). **c** Schematic representation of the IL2RG Pro58Ser and previously reported hypomorphic *IL2RG* mutations denoted (in blue). Signal peptide (SP: positions 1-22) and domains extracellular (EC: 23-262), fibronectin type III (FN-III): (1): 59-151; (2):154-2462, transmembrane (TM: 263-283) and cytoplasmic: (284-369) (based on NCBI Reference Sequence: NP_000197.1 and UniProtKB- P31785). **d** Structure of *Homo sapiens* IL-2 cytokine receptor complex (Protein Data Bank accession number 2b5i). Complex contains 4 protein chains; IL-2 (magenta), IL2RG (cyan), and IL2RA and IL2RB (both gray). The Pro58 residue in IL2RG highlighted in red and Ser58 mutation in orange

DNA samples were extracted from peripheral blood and lymphocyte subpopulations sorted using standard methods. Whole-exome sequencing (WES) was performed on HiSeq 1500 Rapid run (Illumina) using a SureSelect Clinical Research Capture Exome kit (Agilent). The sequencing and data analysis were performed as previously described [20, 21]. Validation of the candidate disease-causing variant and screening of familial mutations in the available family members (Fig. 1a) were conducted using PCR amplification of genomic DNA and capillary electrophoresis using the ABI-3730XL DNA Analyzer and BigDye Terminator Cycle Sequencing kit (Applied Biosystems). Lymphocyte subpopulation screening for the variant was performed using PCR amplification of the genomic DNA and capillary electrophoresis using the cycle sequencing technology (dideoxy chain termination/cycle sequencing) on ABI-3730XL DNA Analyzer. Detailed processing and analysis of WES along with details for the second patient are described in the Online Resource Supplementary text.

### Cell isolation, surface staining, and basic immunological workup

Cell isolation is described in the Online Resource Supplementary text. Peripheral blood mononuclear cells (PBMCs) were stained with fluorescently conjugated anti-human CD4, CD19 (BioLegend), CD3, CD14 (ImmunoTools), CD16, CD56 (BD Pharmigen), and CD8 (Miltenyi Biotech) antibodies for 30 min on ice. After surface staining, SYTOX Green Dead Cell Stain (Invitrogen) was added to the cells, and CD4+ and CD8+ T cells, CD19+ B cells, and CD16+CD56+ NK cells were sorted with BDInflux. Basic immunological workup was performed in an accredited laboratory. Whole-blood NK cell phenotyping and TCRVβ repertoire sequencing are described in the Online Resource Supplementary text.

Expression of IL2RG (CD132) and IL2RA (CD25) was determined from CD4+ T cells using fluorescently conjugated anti-human CD4, CD8, CD25, CD56 (BD Biosciences), and CD132 (eBioscience) antibodies. Briefly, antibodies were added directly to an aliquot of 100 μl of freshly drawn whole blood, pre-cooled to + 4 °C. After 15-min incubation, red blood cells were lysed (BD FACS Lysing Solution) and cells were analyzed by flow cytometry (NovoCyte model 3000 and NovoExpress, Acea).

### STAT Phosphorylation in Response to Exogenous IL-2 and IL-21

STAT5 and STAT3 phosphorylation were measured from isolated PBMCs after a 15-min stimulation in the presence of exogenous IL-2 (10 U/ml and 320 U/ml) or IL-21 (10 ng/ml), respectively, in pre-warmed RPMI 1640. IL-2-stimulated cells were then fixed and permeabilized according to manufacturer’s protocol (Becton Dickinson) and stained with fluorescent-conjugated CD3 (Invitrogen), CD4, CD25, CD56 (BD Biosciences), and pSTAT5 (eBioscience) antibodies. Cells were analyzed by flow cytometry (NovoCyte model 3000 and NovoExpress software, Acea). IL-21 stimulated cells were stained with antibodies for CD4, CD8, and CD19 (BioLegend) for 20 min on ice and fixed with 4% formaldehyde (Thermo) for 10 min. After washing, cells were incubated in 90% methanol at − 20 °C overnight and stained with fluorescent conjugated anti-CD3 (BD), anti-pSTAT3 (Y705, BD), and anti-STAT3 (BD). Cells were analyzed using BD Fortessa flow cytometer and FloJo (v10.6) software.

### Quantitative PCR

CD4+ cells were purified from PBMCs by negative selection with the human CD4+ isolation kit (Miltenyi Biotec) according to the manufacturer’s instructions. Cells were incubated in RPMI 1640 medium (Gibco, Thermo Fischer Scientific) supplemented with 0.5% FBS (HyClone), 2 mM L-glutamine and 0.1 mg/ml penicillin-streptomycin (Gibco, Thermo Fischer Scientific); 10^6^ cells/well at + 37 °C for 2 h prior to IL-2 stimulation (100 U/ml, Sigma). After 4-h stimulation, cells were pelleted and snap-frozen to be further processed. Both stimulated and unstimulated conditions were performed in triplicates for each sample. RNA was extracted using Qiagen RNeasy Mini kit according to the manufacturer’s instructions. A total of 240 ng of RNA was used as a template for cDNA synthesis (Bio-Rad iScript cDNA synthesis kit according to the manufacturer’s instructions). Quantitative PCR (qPCR) in technical triplicates was performed using Taqman protocol and probes according to the manufacturer’s instructions and analyzed with BioRad CFX Touch Real-Time system. The used Taqman probes are listed in the Online Resource Supplementary text. Results were analyzed with GraphPad Prism 7.03 software.

### Blast Formation and Lymphocyte Proliferation in Response to Exogenous IL-2

Blast formation was studied by flow-cytometric assay for specific cell-mediated immune-responses in activated whole blood (FASCIA). Heparinized blood was diluted in RPMI medium containing Glutamax I, gentamicin, and β-mercaptoethanol and stimulated with IL-2 (R&D Systems) in concentrations of 40 U/ml and 320 U/ml for 5 days. After stimulation, cells were stained with Multitest 6-Color TBNK reagent (BD) including CD3, CD45, CD4, CD8, CD19, and CD16/CD56 antibodies. After red blood cells were lysed with FACS lysing solution (BD), lymphocyte subpopulations were analyzed for the percentage of blasts based on their light scatter characteristics using flow cytometry (NovoCyte; Acea). In studying lymphocyte proliferation, purified PBMCs were incubated in culture medium (2 × 10^5^ cells per well) in 96-well polystyrene plates in the presence of increasing concentrations of IL-2 (R&D Systems) for 72 h. For the last 6 h, 1 μCi of 3H-Thymidine was added to each well. All conditions were performed in triplicates. Cells were harvested using a cell harvester (Tomtec) and thymidine incorporation was measured with beta-scintillation counter (MicroBeta; Perkin Elmer).

### IL2RG Expressing Flp-In 293T-REx Cell Lines

Pro58Ser-mutant plasmid was created using Quick-Change Site-Directed Mutagenesis kit (Agilent Technologies) to wild-type *IL2RG* obtained from the human ORFeome collection. Constructs were further subcloned into C-terminal MAC-tag gateway destination vector [22]. The Flp-In 293T-REx cells (Invitrogen) were cultured as instructed by the manufacturer. The constructs were transfected into cells with FuGENE HD Transfection Reagent (Promega) together with pOG44 Flp recombinase expression vector (Thermo Fisher Scientific). The cells were grown under selection with hygromycin B to create a stable cell line.

### Affinity Purification and Mass Spectrometry

Affinity purification of BioID and liquid chromatography-mass spectrometry (LC-MS), analyses were performed as previously described [22]. In BioID-method, a modified biotin ligase is fused to the protein of interest, which enables the biotinylation of the proteins that are proximal. The biotinylated closely interacting proteins are then affinity-purified and analyzed with quantitative mass spectrometry [23]. Briefly, for each pull-down, approximately 5 × 10^7^ cells (5 × 15 cm dishes) in two biological replicates were induced with 2 μg/ml tetracycline and 50 μM biotin (Sigma) for 24 h. Cells were harvested, pelleted, and lysed in HENN-lysis buffer (HENN; 50 mM HEPES pH 8.0, 5 mM EDTA, 150 mM NaCl, 50 mM NaF, supplemented with 0.5% NP40, 1 mM DTT, 1.5 mM Na_3_VO_4_, 1 mM PMSF, and 1x protease inhibitors cocktail; Sigma). Affinity purification was done using Strep-Tactin beads (IBA GmbH). Bound proteins were eluted with freshly prepared D-biotin (Thermo Fisher Scientific). The sample proteins were reduced with TCEP (Tris(2-carboxyethyl)phosphine), alkylated with iodoacetamide, and digested with trypsin (Promega).

LC-MS analysis was done with a Q Exactive ESI-quadrupole-orbitrap mass spectrometer coupled to an EASY-nLC 1000 nanoflow LC (Thermo Fisher Scientific) using the Xcalibur version 3.1.66.10 (Thermo Fisher Scientific). For each sample, two technical replicates were analyzed.

### Database Analysis

SEQUEST search algorithm in Proteome Discoverer software (Thermo Fisher Scientific) was used for peak extraction and protein identification with the human reference proteome of UniProtKB database (www.uniprot.org, Uniprot human 10_2016, with 20,118 sequences). Allowed error tolerances were 15 ppm and 0.05 Da for the precursor and fragment ions, respectively. Database searches were limited to fully tryptic peptides allowing two missed cleavage and carbamidomethyl +57,021 Da (C) of cysteine residue was set as fixed, and oxidation of methionine +15,995 Da (M) as dynamic modification. For peptide identification, false discovery rate was set to < 0.05. The high-confidence protein-protein interactions were identified using stringent filtering against green fluorescent protein control samples. The bait normalized relative protein abundances (% to the IL2RG) were calculated from the spectral counts. Statistical difference was calculated with Student’s *t* test. Gene ontology (GO) annotations were obtained from DAVID bioinformatics resources [24, 25].

## Results

### Case Report

An 11-year-old boy with normal (-1SD) growth was born to nonconsanguineous Finnish parents (Fig.1a). At age two, he started having recurrent upper and lower respiratory tract infections, prolonged cough, and bilateral purulent middle ear infections. At age six, tympanostomy was performed. However, recurrent middle ear infection and purulent discharge from tympanostomy tubes continued and bronchiectasis was noted (Fig.1b), with cystic fibrosis and ciliary dyskinesia ruled out. After two bouts of post-infectious synovitis in the knee at ages eight and nine, HLA-B27, rheumatoid factor, cyclic citrullinated peptide antibodies, and antinuclear antibodies were tested negative.

The patient’s T, B, and NK cell counts were within the normal range, with elevated γδ lymphocyte counts comprising 35.9% of CD3+ T cells. Both CD4+ and CD8+ effector memory (TEM) populations were decreased, with almost absent CD4+ terminally differentiated effector cells (TEMRAs). Regulatory T cell (T_reg_) numbers were normal. Numbers of circulating CD27+ B memory and switched memory B cells were very low. Plasmacytoid dendritic cells (DCs) comprised only 0.02% of the total leukocytes (Table S1, Online Resource). NK cell phenotyping showed that patient had increased amount of CD3-CD56^bright^ NK cells when compared to healthy controls. Furthermore, these cells expressed highly CD27 and inhibitory receptor NKG2A, while CD57 expression was lower than in controls (Table S2, Online Resource). TCRVβ repertoire was comparable to healthy controls (Table S3, Online Resource).

The patient mounted normal antibody responses to tetanus and pneumococcal vaccines, antibodies against *Haemophilus influenzae* and diphtheria reached protective levels only after additional booster doses. Proliferative responses to mitogens (concanavalin A, phytohemagglutinin, pokeweed) have fluctuated (Table S1, Online Resource).

Patient’s plasma immunoglobulin levels have remained normal. Due to recurrent infections and bronchiectasis, azithromycin prophylaxis, and inhalations with hypertonic saline, fluticasone and salbutamol were started at age seven. As recurrent infections continued, intravenous immunoglobulin (IVIG) treatment was started at age nine. The frequency of infections reduced without evident progression of bronchiectasis or his clinical condition. For more detailed case report, see Supplementary text, Online Resource.

### Genetic Analysis

WES identified a novel c.172C>T hemizygous missense variant in the *IL2RG* gene (ENSG00000147168:ENST00000374202: c. 172C>T;p.(Pro58Ser)). Proline-58 is conserved and located in the extracellular part of the IL2RG, close to the fibronectin-III domain (Fig. 1c, d). The screening of the patient’s mother implicated a de novo mutation (Fig. 1a and S1, Online Resource). Analysis of the WES data did not reveal any other compelling pathogenic or likely pathogenic variants in genes that could contribute to the phenotype based on current knowledge (Tables S4 and S5, Online Resource). Sequencing of the patient’s different lymphocyte subsets (CD4+, CD8+, CD19+) and CD16+CD56+ NK cells confirmed the presence of the mutation in all these (Fig.S1, Online Resource).

### Reduced IL2RG Expression on Cell Surface Affects IL-2, IL-15, and IL-21-Mediated Signal Transduction

**Fig. 2:**
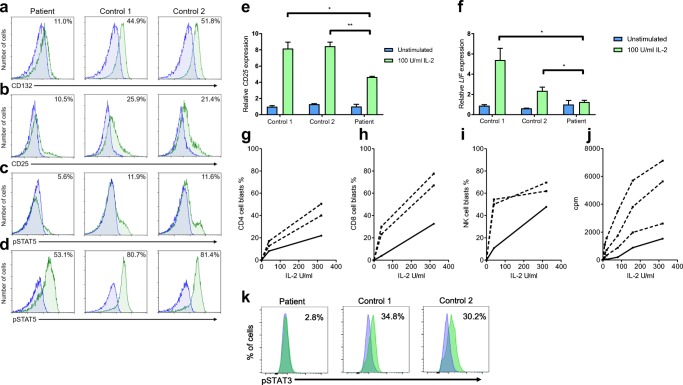
IL-2 and IL-21 responses are disturbed in patient lymphocytes. Expression of **a** IL2RG (CD132) and **b** IL2RA (CD25) on patient’s CD4+ T cells, and phosphorylation of STAT5 in CD4+ T cells in response to IL-2 stimulation at **c** low (10 U/ml) and **d** high (320 U/ml) concentration. Representative histograms for the patient (left) and two control subjects (middle, right) are shown in green as an overlay together with FMO staining (blue). **e***CD25* and **f** leukemia inhibitory factor (*LIF*) mRNA expression after 4-h stimulation with IL-2 (100 IU/ml) determined by quantitative PCR. Represented as fold change (patient unstimulated condition normalized as 1). **g** CD4+ T cell, **h** CD8+ T cell, and **i** CD56+ NK cell blast formation in response to 40 U/ml and 320 U/ml of IL-2 by FASCIA. **j** In vitro lymphocyte proliferation in response to various concentrations of IL-2. PBMC were stimulated for 72 h with 6 different concentrations of recombinant IL-2 in a 3H-Thymidine incorporation assay. Curves represent proliferation (in cpm) of patient’s cells (solid line) and of three healthy controls (dotted lines) all tested in parallel. **k** Phosphorylation of STAT3 in CD3-CD19+ B cells in response to IL-21 (10 ng/ml) shown in green as an overlay with unstimulated cells (blue). Data are representative out of three independent experiments in **a**–**d**, and out of two in the rest. * = *p* < 0.05; ** = *p* < 0.01, determined by unpaired *t* test with Welch’s correction in **e**–**f**

In order to study if the Pro58Ser amino acid substitution in IL2RG affects IL-2R expression on patient lymphocytes, we stained patient’s and healthy donors’ lymphocytes with antibodies specific for IL2RG (CD132) and IL2RA (CD25) and analyzed their surface expression by flow cytometry. In the patient, we found a clear reduction in both IL2RG and IL2RA surface expressions on CD4+ T cells when compared to healthy donors (Fig. 2a, b). Furthermore, we observed similar trends of lower IL2RG surface expression in patient’s CD8+ T and CD56+ NK cells (Table S6, Online Resource). In order to study whether the reduced surface expression was due to lower *IL2RG* gene in expression in patient cells, we performed qPCR on CD4+ T cells. The expression of *IL2RG* mRNA in the patient’s CD4+ T cells was at an equal or slightly increased level compared to our healthy controls (Fig.S2, Online Resource), indicating that Pro58Ser mutation may affect protein stability or targeting to cell surface.

To study whether signaling through IL-2R was impaired due to the mutated *IL2RG*, we studied STAT5 phosphorylation in response to IL-2. At low IL-2 concentrations (10 U/ml), STAT5 phosphorylation was evident on 5.6% of patient CD4+ T cells compared with 11.9% and 11.6% (mean 11.8%) of CD4+ T cells in the controls (Fig. 2c). To assess if higher IL-2 concentration can compensate the signaling defect, we simulated patient and healthy control cells with 320 U/ml of IL-2, which induced STAT5 phosphorylation in 53.1% of CD4+ T cells in the study subject, compared to STAT5 phosphorylation in 80.7% and 81.4% (mean 70.3%) of CD4+ T cells in healthy controls (Fig. 2d). Data indicate that the remaining IL-2R on the patient’s CD4+ T cell surface was insufficient to mediate proper IL-2-induced STAT5 phosphorylation.

Next, we studied if reduced STAT5 phosphorylation affected the expression of known IL-2 target genes. After a 4-h stimulation with 100 U/ml IL-2, expression of *CD25* was significantly lower in the patient cells compared to healthy donors (approximately 4.5-fold induction in the patient vs. 8-fold induction in controls when compared to unstimulated conditions) (Fig. 2e). In response to IL-2 stimulation, leukemia inhibitory factor expression stayed at basal level in patient cells but increased significantly in controls (Fig. 2f).

With FASCIA, lymphocyte blast formation in response to varying IL-2 concentrations (40 U/ml and 320 U/ml) was clearly impaired in index patient’s T (Fig. 2g, h) and NK (Fig. 2i) cells compared to healthy controls. His PBMCs also showed very weak proliferation responses to low concentrations of IL-2 (3 to 13% of the level in healthy controls at 10 U/ml) (Fig. 2j). At higher IL-2 concentrations (up to 320 U/ml), an increasing proliferation response was detected in his cells (Fig. 2j) but remained lower than in controls (21 to 58% of the level in healthy controls at 320 U/ml of IL-2). With hypomorphic mutations of IL2RG, higher IL-2 concentrations could theoretically enhance signaling through IL-2R. However, compared to controls, high IL-2 concentrations had no relative effect on the STAT5 phosphorylation or proliferation. These data indicate that signaling through IL-2 receptor was impaired in patient lymphocytes.

Since IL2RG is also a component of IL4, IL-15, and IL-21 receptors, signal transduction for these cytokines was also studied [4]. We found that STAT3 phosphorylation in patient’s B cells was clearly reduced in response to 10 ng/ml of IL-21 stimulation, compared to healthy controls (Fig. 2k). However, in T cells, we observed insignificant differences in STAT3 phosphorylation between the patient and controls (data not shown). An significant trend towards lower STAT6 phosphorylation in response to IL-4 stimulation in patient CD4+ T cells was noted, but it was normal in CD8+ T and CD19+ B cells (data not shown). STAT5 phosphorylation in response to IL-15 was normal in patient’s lymphocytes (data not shown). In studying IL-15 responses by FASCIA, NK cells behaved normally, whereas blasting of CD4+ and CD8+ T cells was reduced (Fig. S3, Online Resource).

### Pro58Ser-Mutation Disturbs IL2RG Plasma Membrane Targeting

**Fig. 3:**
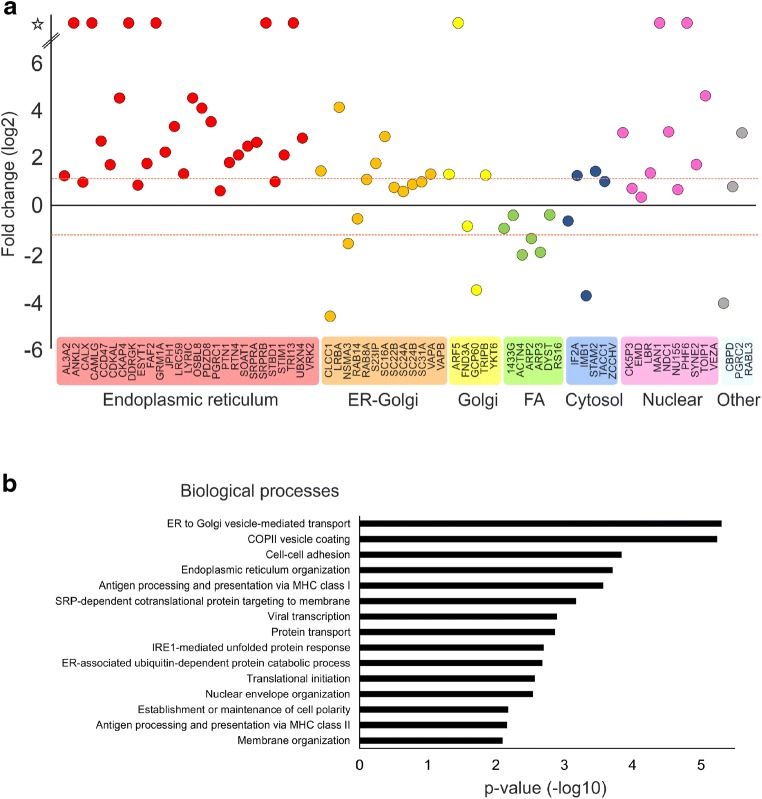
Pro58Ser IL2RG mutant displays increased interactions with ER/Golgi and nuclear proteins. BioID analysis of Pro58Ser mutant- and WT IL2RG. **a** Interacting proteins with a significant differences (*p* < 0.05) between Pro58Ser-mutant and WT IL2RG. The mean fold changes are calculated as label-free MS/MS abundances of Pro58Ser/WT IL2RG. The interactors are classified based on cellular localization. Red dashed line indicates more than two times change on the interacting protein abundance in Pro58Ser mutant compared with the wild type. FA = focal adhesion; star = no detected in WT IL2RG (on/off-differences). **b** Interacting proteins that differ between Pro58Ser and WT were categorized according to their involvement in biological processes (Gene Oncology, Biological Processes terms) via DAVID bioinformatics resources

To characterize why the Pro58Ser mutation affects the cell surface expression of IL2RG, we analyzed the interaction profiles of wild-type (WT) IL2RG and Pro58Ser IL2RG in inducibly expressing HEK293 cell lines using BioID proximity labeling [22]. BioID-analysis showed 69 significantly (*p* < 0.05) altered protein-protein interactions between Pro58Ser-mutant and WT IL2RG (Table S7, Online Resource). Localization-based classification of these altered interacting proteins revealed a marked change in IL2RG localization; mutant IL2RG had increased interactions with ER/Golgi and nuclear proteins, whereas interactions with the proteins localized in focal adhesion were clearly diminished (Fig. 3a). In addition, a GO analysis for their biological processes showed that mutation significantly increased interactions with proteins involved in many ER-Golgi-associated functions, such as ER to Golgi vesicle-mediated transport and COPII vesicle coating (Fig. 3b). These results suggest abnormal maturation of mutant IL2RG, which causes the failure of the plasma membrane targeting and the mislocalization of mutant IL2RG to the ER-Golgi interface.

## Discussion

We report a novel *IL2RG* p.(Pro58Ser) mutation causing X-CID and provide evidence for the impaired IL-2 responses seen in the patient being caused by increased retention of the mutated protein in ER and Golgi. Our patient’s CD4+ cells, compared to healthy controls, showed reduced surface expression of IL2RG and IL2RA (CD25), leading to impaired STAT phosphorylation responses to IL-2 and IL-21 (Fig. 2). Reduced or absent cell surface expression of IL2RG, caused by defect in the transcriptional level or by protein instability, has been reported in hypomorphic IL2RG mutations [13, 26–30]. Direct effect of IL2RG mutation on CD25 surface expression has been previously demonstrated in EBV-transformed cell lines [31] and CD34+ hematopoietic progenitor cells [32] of X-SCID patients along with one additional case termed as atypical X-SCID in which both absolute CD3+ counts and mitogen responses fluctuated so that unambiguous categorization was rendered impossible [33]. The altered amino acid residue resides in the extracellular domain of IL2RG (Fig. 1c, d); theoretically, this could also affect its ligand-binding affinity, but this remains to be studied.

During the revision process of this article, another boy detected by newborn screening for SCID with identical mutation came to our attention. He had low but not undetectable amounts of TRECs and presented with moderately decreased CD4+ and normal B, CD8+ and NK cell counts (Table S8, Online Resource). He is currently 3 years old without signs of illness. A patient with late-onset CID and probable revertant somatic mosaicism with a different mutation in the same position (c. 172C>A, p.(Pro58Thr)) has also been reported [12]. This patient had decreased IgA and IgG levels, occasional swelling of the knee and elbow joints, recurrent interstitial pneumonia, and watery diarrhea. He died of graft failure after an unsuccessful hematopoietic stem cell transplantation (HSCT).

The high-affinity heterotrimeric IL-2R receptor consists of IL2RA (CD25), IL2RB (CD122), and IL2RG (CD132) [34]. Reduced expression of any of the receptor components may affect IL-2 signaling [35–37]. IL2RG participates in signaling of interleukins 2, 4, 7, 9, 15, and 21 [4–7]. Impact of *IL2RG* mutations on various IL2RG-dependent pathways varies; hierarchies in the cytokine signaling impairment have been shown with some IL2RG-dependent signaling pathways more or less impaired than others [26, 29, 38, 39]. In our patient, IL-2 and IL-15 signaling in T and IL-21 signaling in B cells were impaired, while IL-4 signaling was insignificantly affected (Fig. 2c–k and Fig. S3b-c, Online Resource). Like in the boy caught by SCID screening, hypomorphic *IL2RG* mutations frequently present with preserved or mildly decreased T and NK cells [27, 39–41]. The moderate functional defects and normal numbers of T, B, and NK cells in the patient also suggest partly retained signaling (Table S1). IL-2 is required for the sustained expansion of T cell populations and promotes the proliferation and survival of T cell receptor (TCR)-activated T cells [6]. It influences effector T cell differentiation and promotes fate decisions in antigen receptor-activated T cells [34]. Reflecting disturbed IL-2 signaling, our patients displayed reduced cell proliferation in response to IL-2 and low numbers of CD4+ TEMs and TEMRAs together with low CD8+ TEMs and T central memory cells (Fig. 2j, Tables S1, 8, Online Resource). IL-2 is also critical for the development of T_regs_ in the thymus and for their maintenance and function in the periphery; index patient’s normal numbers of T_regs_ and the lacking severe autoimmunity further confirmed hypomorphism (Table S1).

IL-21 and IL-4 are produced and secreted by T follicular helper cells (T_FH_) [8, 42]. IL-21 supports the differentiation and survival of B cells and is required for germinal center reactions, production of plasma cells and IgG class switch recombination [43, 44]. IL-4, in autocrine fashion, enables T_FH_s to activate the activation-induced cytidine deaminase enzyme in B cells, which in turn is necessary for class-switch recombination and affinity maturation [8]. Thus, IL-4 and IL-21 in combination are required for normal B cell function and humoral responses. Besides IL-4 and IL-21, plasma cell differentiation also requires IL-2 [45, 46]. Our patient had high numbers of naïve B cells but low numbers of switched memory B cells, B memory cells, and plasmablasts, likely caused by defective IL2RG plasma membrane targeting and consequently impaired collaborative IL-2 and IL-21 signaling. The patient benefited from IVIG therapy despite apparently normal serum immunoglobulin levels. This may indicate that his endogenous immunoglobulins may not mature into full avidity and/or his B cell memory may not be long-lasting.

The patient also had very low number of plasmacytoid DCs, which could be associated with the defective IL2RG-dependent cytokine signaling; IL-4 and IL-15 are known survival factors for DCs [6]. Although the IL2RG expression was reduced on the surface of patient’s NK cells, their level in peripheral blood has remained normal (Tables S1 and S6) [4, 34]. However, their IL-2-stimulated blast formation was impaired (Fig. 2i), indicating that IL-2 signaling in patient’s NK cells was partly affected by IL2RG Pro58Ser mutation. Patient had increased proportion of CD56^bright^ NK cells, usually considered cytokine expressing immature cells able to become cytotoxic upon appropriate activation (Table S2, Online Resource) [47]. These cells expressed highly the effector marker CD27 (84.1% of CD56^bright^ cells) and the inhibitory receptor NKG2A (71.5% of CD56^bright^ cells) and decreased amounts of CD57, a marker for terminally matured NK cells [48, 49]. In conclusion, patient’s CD56^bright^ NK cells displayed characteristics of both early and later NK cell development stages. Similarly, increased frequencies of CD56^bright^ NK cells have been reported in a patient with CD25 deficiency [50]. Like for the case with CD25 deficiency, our preliminary data suggest that the degranulation ability of both CD56^briht^ and CD56^dim^ cells was restored when patient cells were stimulated with K562 cells in vitro (data not shown).

In increasing numbers of genetic immunodeficiencies, including hypomorphic SCID gene mutations, lymphocyte numbers remain normal despite clear functional defects [51]. Numbers of reported patients carrying such mutations have increased sharply with the increasing use of next-generation sequencing (NGS) early in the diagnostic process. The early use of NGS followed by functional testing of novel mutations enables expansion of the initially described, typically very severe phenotypes, and thus greatly facilitates the diagnosis and targeted treatment of CID patients. In Table 1, we briefly review the reported, putatively hypomorphic *IL2RG* mutations [11, 13, 26–29, 38–41, 52–55]. Atypical X-SCID cases are frequently also caused by genetic reversion [15–17, 19, 58], which can easily be detected by sequencing of different lymphocyte subpopulations. To exclude maternal engraftment, one may use HLA typing or short tandem repeat (STR) analysis [18, 59, 60]. Frequently in historical reports, somatic mosaicism and/or maternal T or NK cell engraftment have not been excluded systematically or the numbers of CD4+ and CD8+ subsets were provided without information of the overall T cell numbers or the numbers of γδ T cells [11, 26, 38, 39, 53, 55]. In these cases, we considered the mutations as potentially hypomorphic and included them in our review. Of note, reactive expansion of the γδ T cells seems particularly common in atypical X-SCID patients [61].

**Table 1:** Reported patients with putatively hypomorphic *IL2RG* mutations with atypical X-SCID/CID phenotype

Mutation	Number of patients	Onset	CD3+ counts	Susceptibility to infections	Opportunistic infections	Bronchiectasis	Diarrhea	Lymph tissue	T cell responses to mitogen stimulation	T cell responses to other stimuli	S/P-IgG	S/P-IgA	S/P-IgM	S-IgE	Responses to vaccine	SCIG/ IVIG treatment	HSCT	Ref
c.115G>A, p.Asp39Asn) and c.115_116ins (27 bp)	1	9 mo	↓/n	+	n/a	n/a	+	↓	↓	↓	n	n	n	↑	↓	–	–	[41, 52]
c.878T>A, p.Leu293Glu	3	0–12 mo	↓	+	+	+/ -	n/a	↓	↓	n/a	n	n/a	n/a	↑, n/a	↓, n/a	–	–	[11, 53]
c.664C>T, p.Arg222Cys	15	2–12 mo	↓/ n; ↓(CD4+), ↓/ n (CD8+)	+	+/−	+/−, n/a	±, n/a	n/↑, n/a	n/↓ n/a	n/↓	n/↓, n/a	n/↓, n/a	n/↓, n/a	↑, n/a	↓, n/a	+	+	[26, 27, 38, 40, 54]
c.485T>G, p.Leu162Arg	1	3	↓	+	+	n/a	n/a	n/a	↓	↓	↓	↓	n	↑/n	n/a	+	–	[27]
c.-105C>T	2	2–14 y	n	+	n/a	+/−	n/a	n/a	↓	↓	n/↓	n	↑/n	n/a	↓	+	–	[13]
c. 52delC, p.Leu18Cysfsstop/Leu18Cysfs*6 (HGMD)	1	15 mo	↓	+	–	n/a	+	n/a	n/a	n	n	↓	n	n/a	n/a	–	–	[55]
c.890A>G, p. Glu297Gly	1	2 y	↓	+	+	n/a	+	n/a	n	n	n	↔	↔	n/a	↓	+	+	[29]
c.982C>T, p.Arg328stop	1	16 mo	↓	+	–	+	n/a	↑	n/↓	↓	n	n	n/a	n/a	n	+	–	[28]
c.87delG, p.Asn31MetfsTer12	2	0–6 mo	n/↑	+	+	n/a	+	n/a	↓	n/a	n/↑	↑	↑	n/↑	↓	+	+	[39]
c.172C>T, p.Pro58Ser	2	0–2 y	n/↓	+/−	–	+; n/a	+; n/a	n; n/1	n/↓	↓	n	n/↔	n	n; n/a	↓; n/a	+/−	–	pres. cases

Listed here are the cases that did not meet the criteria for typical SCID (CD3+ count < 300/μl) defined in [56, 57] and either displayed no signs of somatic mosaicism or maternal engraftment or did not address the issue. In one additional case (not listed here), both absolute CD3+ counts and mitogen responses fluctuated so that unambiguous categorization was rendered impossible [33]

n = normal value, ↓ = value below normal range, ↑ = value above normal range, ↔ = borderline value, n/a = not available; reference values author-defined

^a^B cells and NK cells were typically in the normal range with 4 exceptions; 2 individuals with NK↓ [27], 1 individual B↓NK↓ [26] and 1 individual B↑ [38]

A well-known example of hypomorphism in *IL2RG* is the c. 664C>T;p.(Arg222Cys) mutation, so far reported in 18 patients [26, 27, 38, 40, 54]. In different reports, these patients were classified as typical or atypical, with the majority presenting with opportunistic infections or an Omenn-like clinical presentation; vast majority of them were treated with HSCT. Ten cases of Arg222Cys patients were included in Table 1 and eight excluded due to an X-SCID phenotype.

Altogether, 29 patients with atypical, putatively hypomorphic *IL2RG* mutations causing atypical X-SCID (variably named as X-CID) have been reported, including ours. Prevalence of the most common clinical features is presented in Fig. S4 (Online Resource). The most common feature (97%) is susceptibility to infections, severest forms of viral and fungal infections were rare. However, 13 (45%) patients had suffered from infections caused by opportunistic pathogens, four despite normal CD3+ T cell counts [27, 40]. Reported inflammatory conditions were surprisingly rare in reviewed patients carrying hypomorphic *IL2RG* mutations, with only three patients suffering from eczema or other skin rashes [11, 26, 38], two from inflammatory arthritis [39], two from interstitial lung disease [38], and one from inflammatory bowel disease [29].

Including our patients, 12 (41%) patients presented with normal plasma/serum immunoglobulin levels (IgG normal in all; IgM, IgA and IgE variable). Interestingly, eight (28%) of the reviewed cases showed similarly skewed B cell subpopulations with increased numbers of naïve cells and/or decreased switched memory cells and plasmablasts as our index patient [26, 29, 38, 39]. Despite constantly normal immunoglobulin levels, our index patient’s clinical condition improved significantly after the start of IVIG treatment. Of the reviewed patients, 16 (55%) had received HSCT and 10 (34%) immunoglobulin replacement, some prior to HSCT. Currently, choosing treatment for X-CID is difficult. Long-term complications and prognosis of combined immunodeficiencies remain unknown. In the future, X-CID patients should be recruited to international registries and follow-up studies to collect information in order to outline treatment options and protocols.

## Electronic Supplementary Material

ESM 1 Figure S1 Sanger sequencing validation of mutation. From available family members (a) and index patient’s lymphocyte subpopulations (b); mutation marked with arrowhead. Figure S2 Relative *IL2RG* mRNA expression in CD4+ T cells from patient and two healthy donors. Patient’s mean normalized as 1. Error bars represent standard deviation from three biological replicates. ** = p <0.01, determined by unpaired t test with Welch's correction, ns = nonsignificant. Figure S3 NK cell, CD4+ and CD8+ T cell blast formation in response to IL-15 stimulation. a) NK cells, b) CD4+ cells, c) CD8+ cells. Dashed line = controls, solid line = index patient. Representative of two independent experiments. Figure S4 Clinical features associated with hypomorphic *IL2RG* mutations. Prevalence (%) of the most common clinical features in the reported patients carrying putatively hypomorphic *IL2RG* mutations (DOCX 13.2 kb)

ESM 2 (PPTX 2.21 mb)

ESM 3 (DOCX 70.4 kb)

ESM 4 (XLsX 51.4 kb)
