# Supplementary material for: Novel Hemizygous IL2RG p.(Pro58Ser) Mutation Impairs IL-2 Receptor Complex Expression on Lymphocytes Causing X-Linked Combined Immunodeficiency
Source: J Clin Immunol. 2020 Feb 19;40(3):503–14. doi: 10.1007/s10875-020-00745-2 (PMC7142052; doi:10.1007/s10875-020-00745-2)
Supplement: Supplementary file 1 — Figure S1 Sanger sequencing validation of mutation. From available family members (a) and index patient’s lymphocyte subpopulations (b); mutation marked with arrowhead. Figure S2 Relative IL2RG mRNA expression in CD4+ T cells from patient and two healthy donors. Patient’s mean normalized as 1. Error bars represent standard deviation from three biological replicates. ** = p <0.01, determined by unpaired t test with Welch's correction, ns = nonsignificant. Figure S3 NK cell, CD4+ and CD8+ T cell blast formation in response to IL-15 stimulation. a) NK cells, b) CD4+ cells, c) CD8+ cells. Dashed line = controls, solid line = index patient. Representative of two independent experiments. Figure S4 Clinical features associated with hypomorphic IL2RG mutations. Prevalence (%) of the most common clinical features in the reported patients carrying putatively hypomorphic IL2RG mutations (DOCX 13.2 kb) [file 10875_2020_745_MOESM1_ESM.docx]

**Figure captions for Supplementary Figures and Tables**

**Figure S1**
**Sanger sequencing validation of mutation**

From available family members (a) and index patient’s lymphocyte subpopulations (b); mutation marked with arrowhead

**Figure S2
Relative *IL2RG* mRNA expression in CD4+ T cells from patient and two healthy donors.**

Patient’s mean normalized as 1. Error bars represent standard deviation from three biological replicates. ** = p <0.01, determined by unpaired t test with Welch's correction, ns = nonsignificant

**Figure S3
NK cell, CD4+ and CD8+ T cell blast formation in response to IL-15 stimulation.**

a) NK cells, b) CD4+ cells, c) CD8+ cells. Dashed line = controls, solid line = index patient. Representative of two experiments.

**Figure S4
Clinical features associated with hypomorphic *IL2RG* mutations.**

Prevalence (%) of the most common clinical features in the reported patients carrying putatively hypomorphic *IL2RG* mutations

**Table S1
Immunologic characteristics of index patient**

**Table S2
NK cell phenotyping of index patient and two healthy controls**

**Table S3
TCRVβ repertoire**

Overall productive clonality (a) of the TCRVβ repertoire and top five TCRVβ clones presented as amino acid sequences with their productive frequencies (b) in percentages (in brackets)

**Table S4
List of filtered variants** **(pathogenic, likely pathogenic, or unknown significance)**

**Table S5
Germline variant in *IL2RG* gene identified in patient with recurrent respiratory infections and bronchiectasis**

**Table S6
Surface expression of IL2RG (CD132)**

On CD4+, CD8+ lymphocytes, CD4+low monocytes, CD3-CD56- B cells and CD56- NK cells measured by mean fluorescent intensity (MFI)

**Table S7**
**High-confidence protein-protein interactions of IL2RG**

Bait normalized Bio-ID results. The high-confidence protein-protein interactions of WT and P58S-mutant IL2RG. The bait normalized relative protein abundances (% to the IL2RG) were calculated from the spectral counts. The MS-interactome data was filtered against GFP controls

**Table S8**

**Immunological characteristics of patient identified by SCID screening**
