# Supplementary material for: Novel Hemizygous IL2RG p.(Pro58Ser) Mutation Impairs IL-2 Receptor Complex Expression on Lymphocytes Causing X-Linked Combined Immunodeficiency
Source: J Clin Immunol. 2020 Feb 19;40(3):503–14. doi: 10.1007/s10875-020-00745-2 (PMC7142052; doi:10.1007/s10875-020-00745-2)
Supplement: Supplementary file 2 — (PPTX 2.21 mb) [file 10875_2020_745_MOESM2_ESM.pptx]

## Slide 1
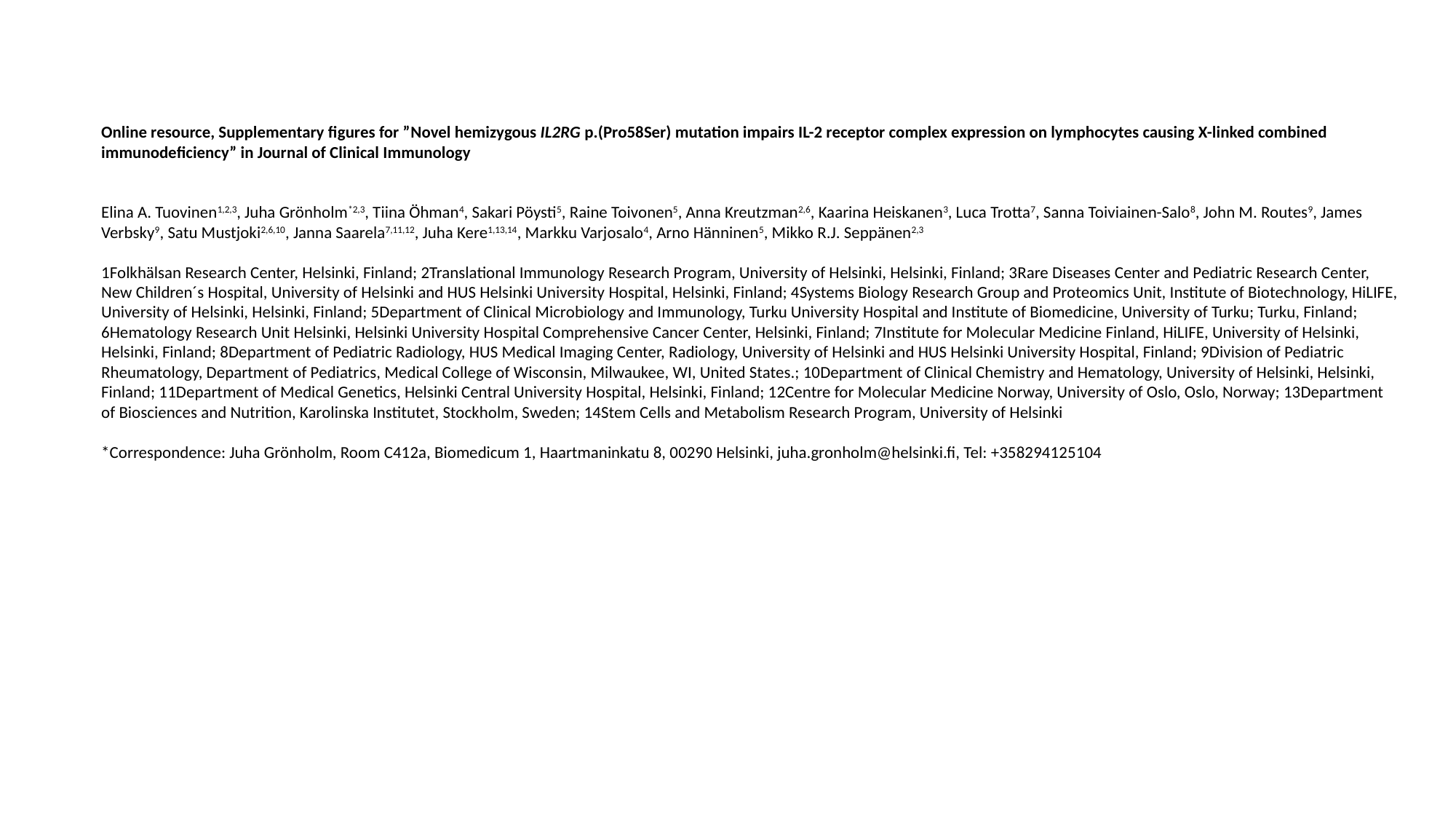

Online resource, Supplementary figures for ”Novel hemizygous IL2RG p.(Pro58Ser) mutation impairs IL-2 receptor complex expression on lymphocytes causing X-linked combined immunodeficiency” in Journal of Clinical Immunology
Elina A. Tuovinen1,2,3, Juha Grönholm*2,3, Tiina Öhman4, Sakari Pöysti5, Raine Toivonen5, Anna Kreutzman2,6, Kaarina Heiskanen3, Luca Trotta7, Sanna Toiviainen-Salo8, John M. Routes9, James Verbsky9, Satu Mustjoki2,6,10, Janna Saarela7,11,12, Juha Kere1,13,14, Markku Varjosalo4, Arno Hänninen5, Mikko R.J. Seppänen2,3
1Folkhälsan Research Center, Helsinki, Finland; 2Translational Immunology Research Program, University of Helsinki, Helsinki, Finland; 3Rare Diseases Center and Pediatric Research Center, New Children´s Hospital, University of Helsinki and HUS Helsinki University Hospital, Helsinki, Finland; 4Systems Biology Research Group and Proteomics Unit, Institute of Biotechnology, HiLIFE, University of Helsinki, Helsinki, Finland; 5Department of Clinical Microbiology and Immunology, Turku University Hospital and Institute of Biomedicine, University of Turku; Turku, Finland; 6Hematology Research Unit Helsinki, Helsinki University Hospital Comprehensive Cancer Center, Helsinki, Finland; 7Institute for Molecular Medicine Finland, HiLIFE, University of Helsinki, Helsinki, Finland; 8Department of Pediatric Radiology, HUS Medical Imaging Center, Radiology, University of Helsinki and HUS Helsinki University Hospital, Finland; 9Division of Pediatric Rheumatology, Department of Pediatrics, Medical College of Wisconsin, Milwaukee, WI, United States.; 10Department of Clinical Chemistry and Hematology, University of Helsinki, Helsinki, Finland; 11Department of Medical Genetics, Helsinki Central University Hospital, Helsinki, Finland; 12Centre for Molecular Medicine Norway, University of Oslo, Oslo, Norway; 13Department of Biosciences and Nutrition, Karolinska Institutet, Stockholm, Sweden; 14Stem Cells and Metabolism Research Program, University of Helsinki
*Correspondence: Juha Grönholm, Room C412a, Biomedicum 1, Haartmaninkatu 8, 00290 Helsinki, juha.gronholm@helsinki.fi, Tel: +358294125104

## Slide 2
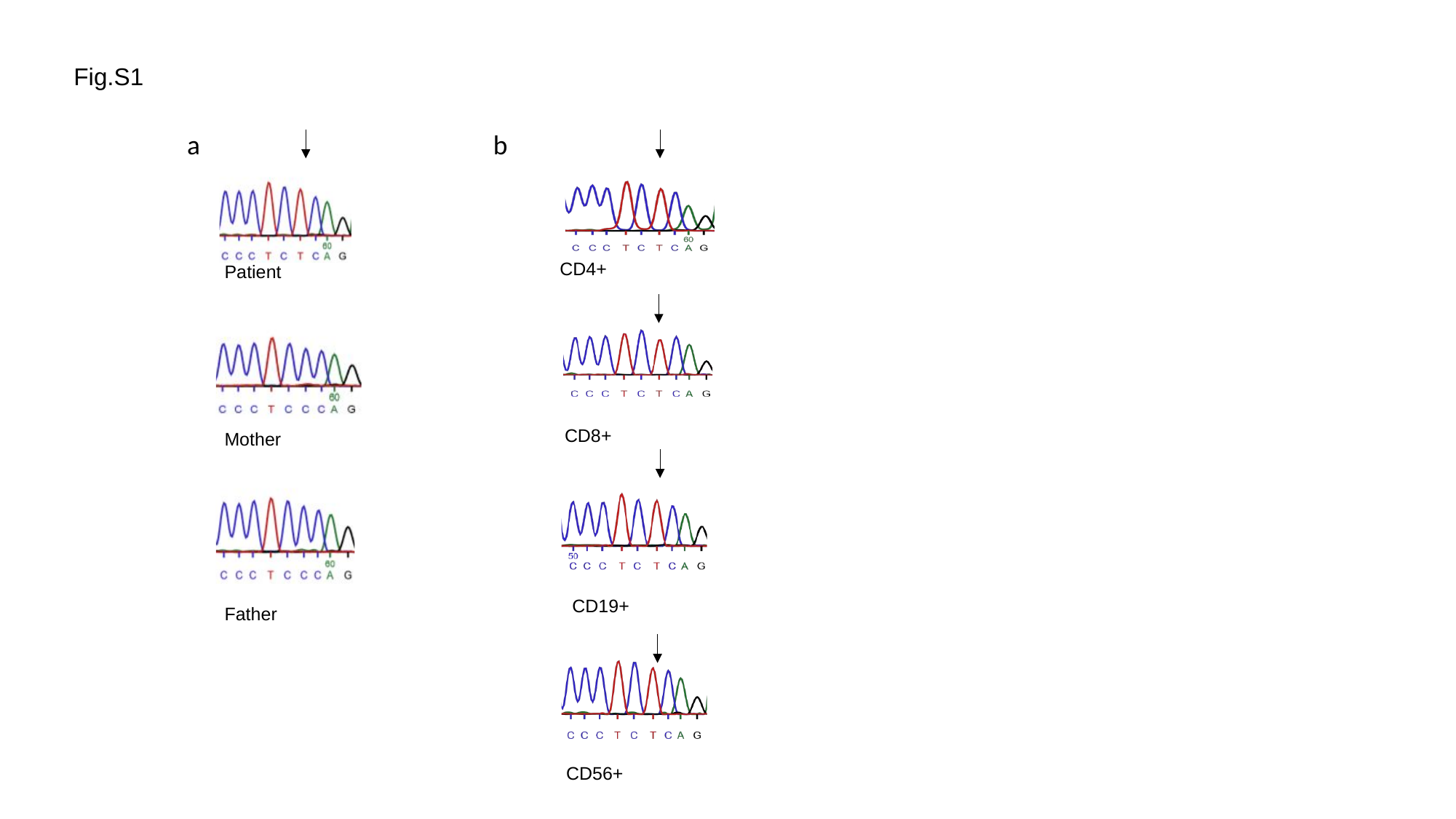

Fig.S1
a
b
CD4+
Patient
CD8+
Mother
CD19+
Father
CD56+

## Slide 3
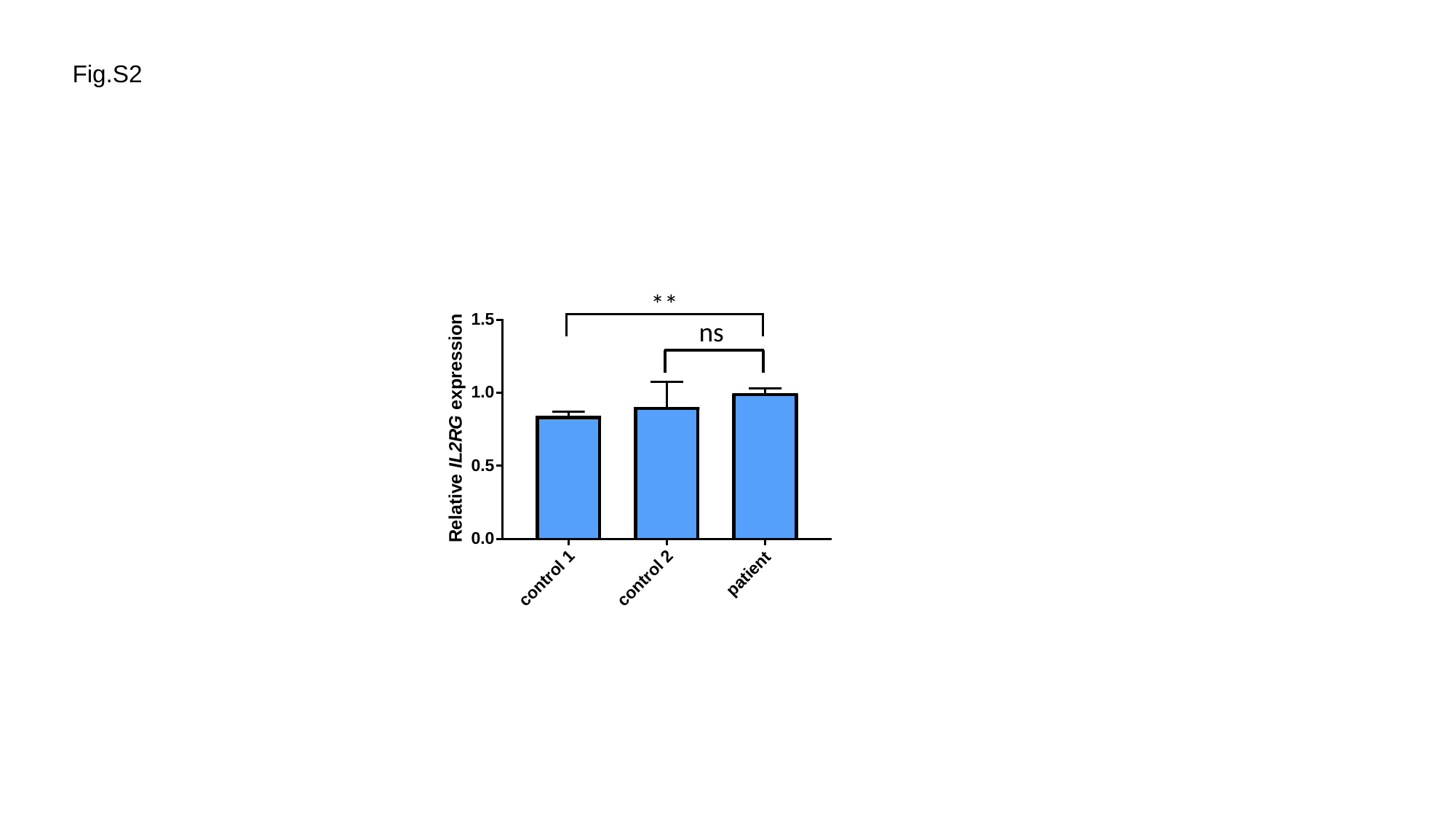

Fig.S2
**
ns

## Slide 4
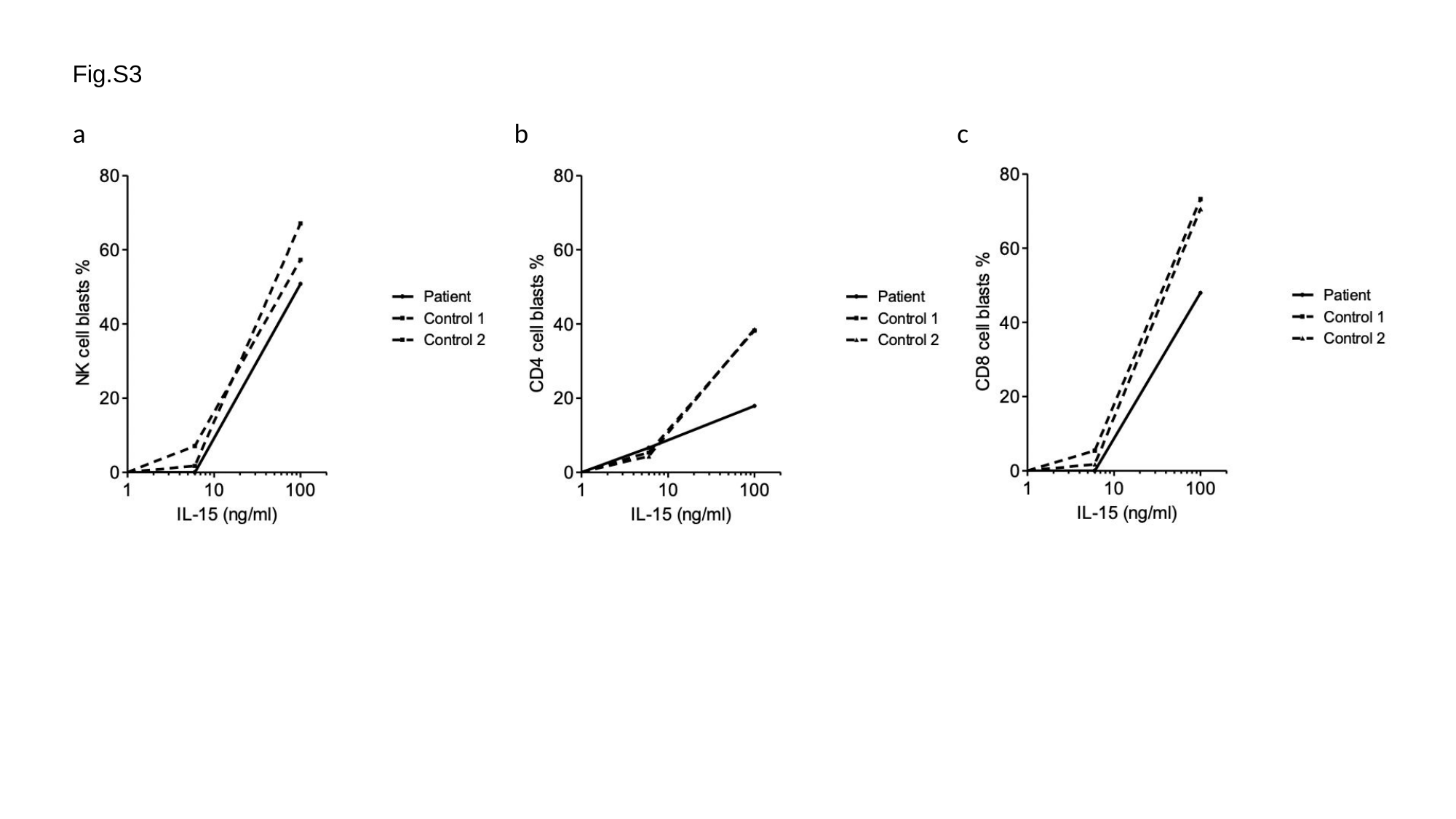

Fig.S3
a
b
c

## Slide 5
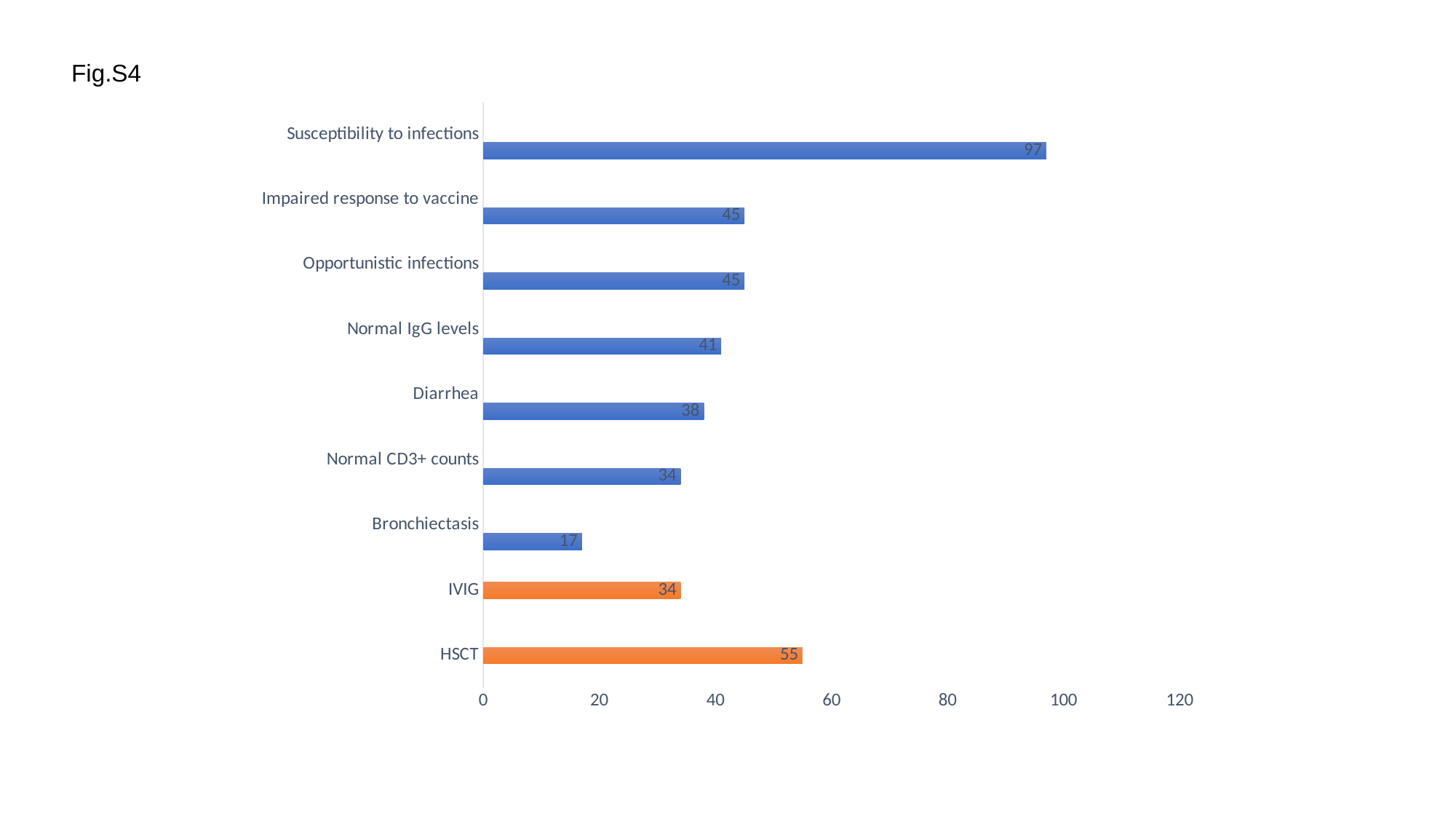

Fig.S4
### Chart
| Category | Sarja 1 | Sarja 2 | Sarja 3 |
|---|---|---|---|
| HSCT | None | 55.0 | None |
| IVIG | None | 34.0 | None |
| Bronchiectasis | 17.0 | None | None |
| Normal CD3+ counts | 34.0 | None | None |
| Diarrhea | 38.0 | None | None |
| Normal IgG levels | 41.0 | None | None |
| Opportunistic infections | 45.0 | None | None |
| Impaired response to vaccine | 45.0 | None | None |
| Susceptibility to infections | 97.0 | None | None |
