# Supplementary material for: Novel Hemizygous IL2RG p.(Pro58Ser) Mutation Impairs IL-2 Receptor Complex Expression on Lymphocytes Causing X-Linked Combined Immunodeficiency
Source: J Clin Immunol. 2020 Feb 19;40(3):503–14. doi: 10.1007/s10875-020-00745-2 (PMC7142052; doi:10.1007/s10875-020-00745-2)
Supplement: Supplementary file 3 — (DOCX 70.4 kb) [file 10875_2020_745_MOESM3_ESM.docx]

**Online Resource Supplementary text**

**Novel hemizygous *IL2RG* p.(Pro58Ser) mutation impairs IL-2 receptor complex expression on lymphocytes causing X-linked combined immunodeficiency
in Journal of Clinical Immunology**

Elina A. Tuovinen^1,2,3^, Juha Grönholm*^2,3^, Tiina Öhman^4^, Sakari Pöysti^5^, Raine Toivonen^5^, Anna Kreutzman^2,6^, Kaarina Heiskanen^3^, Luca Trotta^7^, Sanna Toiviainen-Salo^8^, John M. Routes^9^, James Verbsky^9^, Satu Mustjoki^2,6,10^, Janna Saarela^7,11,12^, Juha Kere^1,13,14^, Markku Varjosalo^4^, Arno Hänninen^5^, Mikko R.J. Seppänen^2,3^

^1^Folkhälsan Research Center, Helsinki, Finland; ^2^Translational Immunology Research Program, University of Helsinki, Helsinki, Finland; ^3^Rare Diseases Center and Pediatric Research Center, New Children´s Hospital, University of Helsinki and HUS Helsinki University Hospital, Helsinki, Finland; ^4^Systems Biology Research Group and Proteomics Unit, Institute of Biotechnology, HiLIFE, University of Helsinki, Helsinki, Finland; ^5^Department of Clinical Microbiology and Immunology, Turku University Hospital and Institute of Biomedicine, University of Turku; Turku, Finland; ^6^Hematology Research Unit Helsinki, Helsinki University Hospital Comprehensive Cancer Center, Helsinki, Finland; ^7^Institute for Molecular Medicine Finland, HiLIFE, University of Helsinki, Helsinki, Finland; ^8^Department of Pediatric Radiology, HUS Medical Imaging Center, Radiology, University of Helsinki and HUS Helsinki University Hospital, Finland; ^9^Division of Pediatric Rheumatology, Department of Pediatrics, Medical College of Wisconsin, Milwaukee, WI, United States.; ^10^Department of Clinical Chemistry and Hematology, University of Helsinki, Helsinki, Finland; ^11^Department of Medical Genetics, Helsinki Central University Hospital, Helsinki, Finland; ^12^Centre for Molecular Medicine Norway, University of Oslo, Oslo, Norway; ^13^Department of Biosciences and Nutrition, Karolinska Institutet, Stockholm, Sweden; ^14^Stem Cells and Metabolism Research Program, University of Helsinki

***Correspondence:** Juha Grönholm, Room C412a, Biomedicum 1, Haartmaninkatu 8, 00290 Helsinki, juha.gronholm@helsinki.fi, Tel: +358294125104

**Extended case report**

The index is a 11-year old male born appropriate for gestational age (3730g, 52 cm) to nonconsanguineous Finnish parents, after an uneventful pregnancy. He received all vaccinations without complications. In infancy and early childhood, the patient suffered from atopic dermatitis, but has not displayed any symptoms or signs of IgE-mediated allergy. While no other prolonged viral infections have been detected, frequently secondarily infected molluscum contagiosum lesions in front of elbows, thighs and abdomen have waxed and waned.

At the age of 18 months, after starting daycare, he developed recurrent upper and lower respiratory tract infections. From the age of two, recurrent acute middle ear infections that developed into acute secretory middle ear infection were noted. Tympanostomy was performed at the age of six, yet recurrent infections and purulent discharge from tympanostomy tubes continued, with *Staphylococcus aureus* in bacterial cultures. The patient experienced pneumonia, at the age of two. Since the age of four, the patient had suffered from prolonged productive cough with suspected poorer physical performance than in his friends. However, at the age of five, impulse oscillometry and chest X-ray were normal.

At the age of seven, the patient presented with ventilation problems during general anesthesia. Bronchoscopy detected mucous discharge with abundant *Haemophilus influenzae* and *Moraxella catarrhalis* growth in bacterial cultures. No other bacteria, viruses or fungi were detected in bronchoalveolar lavage. Carinal biopsy revealed chronic bronchitis with a thickened basal membrane. Electron microscopy of cilia excluded ciliary dyskinesia. Sweat test chloride level (48 mmol/l) was inconclusive, but genetic testing excluded cystic fibrosis. Fecal elastase and serum antitrypsin were normal. HRCT revealed bronchiectasis in both lower lobes, in the medial segment of middle lobe and in the lingula. Spirometry, however, was not suggestive of airway disease. Due to recurrent infections and bronchiectasis, azithromycin prophylaxis and inhalations with hypertonic saline, fluticasone and salbutamol were started at the age of seven. The frequency of infections was somewhat reduced, with improved respiratory symptoms. At repeat HCRT at the age of nine, his bronchiectasis showed no progression, but the lung volumes remained somewhat low. Plasma and serum immunoglobulin levels (IgA, IgG, IgM, IgE) were normal for age. Tetanus and pneumococcal vaccine responses were normal. Antibodies against diphtheria and *Haemophilus* *influenzae* reached protective levels after re-vaccination. Lymphocyte counts are shown in Table S1. The suppression capacity of patient’s Treg cells could not be tested due to non-responsiveness of the cells to anti-CD3/CD28 stimulus. As recurrent infections continued, intravenous immunoglobulin substitution was started at the age of nine with plasma IgG target level of > 9 g/l. Subsequently, the patient has had no infections or symptoms and his spirometry values have normalized.

The patient suffered from toddler diarrhea. Allergy, coeliac disease and lactose intolerance were excluded. Since the age of five he also suffered from constipation, encopresis and coprostasis. Resilient enterobiasis was treated with multiple courses of pyrvinium pamoate and mebendazole. Fecal calprotectin levels have fluctuated between normal and elevated (80-275 µg/g; ref <100 µg/g). Fecal antitrypsin has transiently elevated only once (822 µg/g; ref <268 µg/g). Due to postitive family history of inflammatory bowel disease, esophagogastroduodenoscopy, ileocolonoscopy and capsule endoscopy were performed at the age of seven with normal macroscopic and histological findings. No other pathogens than enterobiasis were found.

The patient has at the ages of eight and nine had two bouts of reactive arthritis in both knees, from which he recovered with courses of NSAIDs. HLA-B27, rheumatoid factor, cyclic citrullinated peptide antibodies and antinuclear antibodies tested negative.

**Whole-exome sequencing**

WES was performed using a SureSelect Clinical Research Capture Exome kit (Agilent, Santa Clara, CA, USA). The sequencing was performed using HiSeq 1500 Rapid run (Illumina, San Diego, CA, USA) as previously described [1,2]. The WES data were analyzed using a version 2.7 of the in-house developed analysis pipeline for quality control and variant identification (VCP) [3]. Raw Illumina reads were first merged with SeqPrep (0.4.5). The resulting paired reads were trimmed of # blocks in the quality scores from the end of the read. After this, any read shorter than 36 base pairs was removed. The paired reads and single reads were aligned separately using the Burrows Wheeler Aligner (version 0.5.10) against the human genome (Ensembl version 70). The alignment was refined using GATK Indel Realignment (version 1.5-3). After the alignment, potential PCR duplicates were removed with Picard MarkDuplicates (version 1.65). Also, any read pair where both reads were mapping or any single reads which was mapping to multiple genomic positions were removed. Variant calling was performed with SAMtools mpileup (version 0.1.18) and dindel (version 1.01). The SNPs were called with minimum depth of 7. The resulting SNPs were then recalculated using quality values. A ratio between sum of quality values of reference (R) and variant (V) calls was calculated: R / (R + V), discarding any where the ratio was above 0.8. If there were two variant calls and no reference, the call with higher quality value was used in place of reference. Any call with ratio smaller than 0.2 was assumed to be homozygous and the rest heterozygous. Annovar was used for the annotations and prediction of functional consequences of the identified variants. All the common (frequencies above 0.01 in the general population) and non-coding variants were discarded. The frequency filtering was based on data from Genome Aggregation Database (gnomAD, Cambridge, MA, USA; http://gnomad.broadinstitute.org/; accessed in May 2017) and the national *SISu* project (<http://sisu.fimm.fi/>) [4,5]. In addition, we discarded all the variants exceeding the frequency of 0.05 according to an internal-use database. The rare variants affecting the coding regions were filtered based on the predicted consequences at the transcript level, with the selection of frameshift, nonsense, splicing and missense variants. All filtered variants were further evaluated according to the ACMG Standards and Guidelines [6]and prioritized using the predicted effect on the protein, the conservation of the affected aminoacids and *in silico* prediction tools. The data were analyzed with an in-house bioinformatics pipeline. We searched for genotypes according to the inheritance patterns (autosomal recessive/dominant or X-linked recessive) possibly occurring in the family.

**Capillary sequencing**

The pinpointed variant in the *IL2RG* gene (Ensembl ENSG00000147168; ENST00000374202; GRCh37.p13), was confirmed using PCR amplifications of genomic DNA and capillary electrophoresis on the ABI-3730XL DNA Analyzer and BigDye Terminator Cycle Sequencing kit (Applied Biosystem, Foster City, CA, USA). The same method was used for the screening of the familial mutations in all the available family members. The sequencing data were analyzed using Sequencher (Gene Codes). The sequences of all the forward and reverse primers used for PCR amplifications and capillary electrophoresis are available upon request.

**Sequencing of the second patient**

Partial exome sequencing was performed using Illumina Trusight kit and MiSeq according to manufacturers instructions. Findings were confirmed by Sanger sequencing, which also confirmed the the patient’s mother as a carrier of the mutation.

**Cell isolation**

T cell phenotyping and activation studies were done simultaneously from the patient (aged 10 years) and 3 healthy adult donors (aged 25 – 55 years) in two separate instances. Blood samples were drawn into lithium heparin vacuum tubes, and until further processed kept at room temperature. For quantitative PCR (qPCR), blood was drawn simultaneously from the patient and two healthy male donors (aged 22 years) and processed as above mentioned. Peripheral mononuclear cells (PBMCs) were isolated using Ficoll paque-gradient centrifugation (Ficoll-Paque, Pharmacia and Ficoll-Paque plus, GE Healthcare).

**NK cell phenotyping**

NK cell subpopulations were immunophenotyped from fresh PB samples from the patient and 2 healthy adult controls for various cell surface markers, including immune checkpoint receptors, markers for chemotaxis, cytotoxicity, and migration. 50 000 CD45^+^ lymphocytes were acquired with FACS Verse (BD) and the data was analyzed with FlowJo software (FlowJo 10.4, FlowJo, LLC 2006-2017).

**TCRVβ repertoire sequencing**

DNA was isolated from 10^6 PBMCs using Flexigene DNA Kit (Qiagen). DNA samples were prepared using Adaptive Biotechnologies immunoSEQ human T-cell receptor beta (hsTCRB) Kit. Sequencing was performed in two biological and technical replicates with MiSeq Reagent Kit v3 150 cycle –kit and Illumina MiSeq. Data-analysis was performed by Adaptive biotech (Immunoseq Analyzer).

**Taqman Probes**

The following Taqman gene-specific probes were purchased from Thermo Fischer Scientific: human *IL2RA* (Hs00907777_m1), human *LIF* (Hs01055668_m1), human *RPL13A* (Hs04194366_g1), human *IL2RG* (Hs00415671_m1). Results were analyzed with GraphPad Prism 7.03 software.

**Table S1** Immunologic characteristics of the index patient

|  |  | **Healthy control median/ reference range (cells/μl)** | **Patient** |
| --- | --- | --- | --- |
| **Leukocytes** |  | **4500-13500/ μl** | **7200/ μl** |
|  | Lymphocytes | 13-48% | 42.6%; 3070/ μl |
|  | Monocytes | 4-9% | 12.5%; 900/ μl |
|  | Neutrophils | 36-77% | 41.0%; 2950/ μl |
|  | Basophils | 0-1% | 1.3%; 90/ μl |
|  | Eosinophils | 0-6% | 2.4%; 170/ μl |
|  | Platelets | 200000-450000/ μl | 302 000/ μl |
| **Dendritic cells ^a^**  **(of lymphocytes)** |  |  |  |
| Plasmacytoid | lin^-^HLA-DR^+^CD123^+^CD11c^-^ | 0.1-0.3% | 0.02% |
| Monocytoid | lin^-^HLA-DR^+^CD123^-^CD11c^+^ | 0.1-0.3% | 0.13% |
| **CD3+ T cells ^a^** |  | **750-2760/ μl; 56-86% (of lymphocytes)** | **1880/** **μl; 65%** |
|  | TCRαβ+ | 88.1-97.8% | 64.1% |
|  | TCRγδ+ | 1.9-11.7% | 35.9% |
|  | Naive CCR7+CD45RA+ | - | 40.2% |
|  | Memory CD45RO+ | - | 38.3% |
|  | CD4+CD8+ | 0.3-3.3 | 0.2% |
|  | CD4-CD8- | 3.1-9.3% | 32.4% |
|  | TCRαβ^+^ CD4-CD8- | - | 1.5% |
| T_reg_ | FOXP3^+^CD25^high^CD127^low^ | 2.8-6.4% | 3.3% |
| **Activated** | HLA-DR+ CD38- | - | 9.8% |
|  | HLA-DR-CD38+ | - | 46.5% |
|  | HLA-DR+ CD38+ | - | 10.5% |
| **CD4/CD8 ratio** |  | 0.8-3.7 | 0.6 |
| **CD3+CD4+ T cells** |  | **404-1612/** **μl; 33-58%** | **503/** **μl;17%** |
| Naive | CCR7+CD45RA+ | 20.5-54.8% | 49.7% |
| TCM | CCR7+CD45RA- | 8.4-32.8% | 43.1% |
| TEM | CCR7-CD45RA- | 19.9-52.4% | 7.0% |
| Temra | CCR7-CD45RA^+^ | 1.4-17.0% | 0.2% |
| T_reg_ | FOXP3^+^CD25^high^CD127^low^ | - | 13.4% |
|  | CD45RA+CD62L+ | 27.4-64.7% | 52.0% |
| Recent thymic emigrants (RTE) | CD45RA+CD62L+CD31+ | 14.4-38.3% | 38.9% |
| Activated | HLA-DR+ CD38- | 2.4-9.6% | 4.6% |
|  | HLA-DR-CD38+ | 40.4-72.9% | 67.1% |
|  | HLA-DR+ CD38+ | 0.9-4.6% | 3.6% |
| **CD3+CD8+ T cells** |  | **220-1130/** **μl; 13-39%** | **860/** **μl; 30%** |
| Naive | CCR7+CD45RA+ | 18.8-71.0% | 61.8% |
| TCM | CCR7+CD45RA- | 1.2-7.3% | 1.1% |
| TEM | CCR7-CD45RA- | 14.6-63.0% | 12.4% |
| Temra | CCR7-CD45RA^+^ | 4.5-33.7% | 24.7% |
| Activated | HLA-DR+ CD38- | 3.8-32.4% | 5.0% |
|  | HLA-DR-CD38+ | 30.3-78.5% | 62.1% |
|  | HLA-DR+ CD38+ | 1.4-21.4% | 6.0% |
| **CD19+ B cells ^b^** |  | **80-620/** **μl; 5-22% (of lymphocytes)** | **500/ μl; 18%** |
| Naive | CD27-IgD+IgM+ |  | 88.2% |
| Memory | CD27+ | 13.0-48.0% | 9.6% |
| Marginal zone-like | CD27+IgD+IgM+ | 5.0-18.0% | 6.7% |
| Switched memory | CD27+IgD-IgM- | 9.0-26.0% | 2.1% |
| Activated | CD38^low^CD21^low^ | 3.0-9.0% | 8.2% |
| Transitional | CD38++IgM++ | 1.0-13.0% | 3.8% |
| Plasmablasts | CD38++IgM- | 1.0-7.0% | 0.3% |
| **CD3+CD16^+^/56^+^** **NK cells** |  | **80-720/ μl; 5-26%** | **480/ μl; 17%** |
| **Serum Immunoglobulins**  **(prior IVIG)** | IgG HUSLAB | 5.6-19.2 g/L | 7.8 g/L |
|  | IgA | 0.35-2.99 g/L | 1.92 g/L |
|  | IgM | 0.36-2.07 g/L | 2.45 g/L |
|  | IgE | 0-320 IU/L | 14 IU/L |
| **Lymphocyte proliferative responses to mitogens** | Phytohemagglutinin (PHA), Concanavalin A (ConA), Pokeweed mitogen (PWM) | | CD4+: PHA↓(slightly decreased), ConA n/↓(decreased), PWM n  CD8+: PHA n, ConA n/↓(decreased), PWM n  CD19+: PWM n/↓ (clearly decreased) |
| **Specific antibodies against vaccine antigens** | Anti-tetanus, anti-pneumococcal  Anti-diphtheria, Anti-haemophilus influenzae | | Tetanus: n; Pneumococcal n;  dipht. ↓; Haemph. ↓ |

When available, in-house (HUSLAB) determined pediatric reference values were used. Other reference values: ^a^HUSLAB reference values for adults; ^b^Piątosa B, Wolska-Kuśnierz B, Pac M, Siewiera K, Gałkowska E, Bernatowska E. B cell subsets in healthy children: Reference values for evaluation of B cell maturation process in peripheral blood. Cytometry 2010;78B(6):372-381

**Table S2** NK cell phenotyping of the index patient and two healthy controls

|  | Control 1 | Control 2 | Patient |
| --- | --- | --- | --- |
| CD3-CD56+ cells (% of lymphocytes) | 20.6 | 18.9 | 12 |
| CD3-CD56bright (% of lymphocytes) | 0.79 | 0.78 | 2.66 |
| CD3-CD56dim (% of lymphocytes) | 19.8 | 18.0 | 9.35 |
| CD3-CD56bright (% of NK cells) | 2.57 | 4.16 | 19.9 |
| CD3-CD56dim (% of NK cells) | 97.3 | 95.3 | 80.1 |
| NKT (CD3+CD56+) bright (% of lymphocytes) | 1.14 | 3.47 | 5.42 |
| NKT (CD3+CD56+) dim (% of lymphocytes) | 2.38 | 4.9 | 1.73 |
| CD3-CD56brightCD27+ | 41.1 | 34.2 | 84.1 |
| CD3-CD56dimCD27+ | 4.78 | 3.3 | 9.22 |
| CD3-CD56bright CD57+ | 25.1 | 49.7 | 3.0 |
| CD3-CD56dimCD57+ | 42.3 | 67.4 | 25.3 |
| CD3-CD56brightNKG2A+ | 57.5 | 9.72 | 71.5 |
| CD3-CD56dimNKG2A+ | 27.7 | 2.58 | 32.0 |
| CD3-CD56brightNKG2C+ | 12.1 | 26.0 | 43.4 |
| CD3-CD56dimNKG2C+ | 2.11 | 10.5 | 13.1 |

**Table S3** Overall productive clonality^a^ of the TCRVβ repertoire and top five TCRVβ clones presented as amino acid sequencies with their productive frequencies^b^ in percentages (in brackets); numbers represented as mean of the two replicates

|  | **Control 1** | **Control 2** | **Patient** |
| --- | --- | --- | --- |
| **Overall productive clonality** | 0.0765 | 0.0988 | 0.0505 |
|  | CASSLRGNEQYF (4.5082) | CASSDYGVSNQPQHF (4.7495) | CASSLARTSGTSYEQYF  (1.6597) |
|  | CAWSVGQSNTEAFF (1.4383) | CASRPLRQGGTEAFF (1.9601) | CASSLASTGNYEQYF  (1.4605) |
|  | CASSLGAGDISGELFF  (1.2961) | CASSYPGSGQGAYNEQFF  (1.0438) | CASDLTGNQPQHF  (1.1404) |
|  | CASKRDRGQYEQYF  (0.3810) | CASSPDRASYEQYF  (0.9411) | CSVGGDGYTF  (0.8731) |
|  | CASRASNQPQHF (0.3726) | CASFDSYYEQYF  (0.8219) | CASSLGGLYEQYF  (0.8009) |

^a^ Clonality measure for the sample calculated over all productive rearrangements. Values for clonality range from 0 to 1. Values near 1 represent samples with one or a few predominant rearrangements (monoclonal or oligoclonal samples) dominating the observed repertoire. Clonality values near 0 represent more polyclonal samples.

^b^Clonality measure for the sample calculated over all Productive Rearrangements. The amino acid translation of the unique nucleotide rearrangement. Only productive rearrangements can be translated. Productive rearrangements are in-frame, do not contain a stop codon and can produce a functional protein receptor. Productive Clonality is calculated by normalizing Productive Entropy using the total number of unique Productive Rearrangements and subtracting the result from 1.

**Table S4** List of the filtered variants (pathogenic, likely pathogenic, or unknown significance)

| **Chr** | **Position** | **Allele** | **Genotype** | **Inheritance** | **Gene** | **Gene id** | **Conse** | **cDNA change^a^** | **Protein change^a^** | **InterVar^b^** | **Frequency^c^** |
| --- | --- | --- | --- | --- | --- | --- | --- | --- | --- | --- | --- |
| chr15 | 73470733 | C/T | He | AD (novel) | *NEO1* | ENSG00000067141 | exonic (nonsynonymous SNV) | c.C284T: | p.A95V | Uncertain significance | 5,70E-05 |
| chr15 | 83518651 | G/C | He | AD (novel) | *HOMER2* | ENSG00000103942 | exonic (nonsynonymous SNV) | c.C881G: | p.A294G | Uncertain significance | 4,48E-05 |
| chr2 | 74042800 | C/A | He | AD (novel) | *C2orf78* | ENSG00000187833 | exonic (nonsynonymous SNV) | c.C1450A: | p.Q484K | Uncertain significance | 7,72E-05 |
| chr3 | 75781257 | T/G | He | AR (compound heterozygote) | *ZNF717* | ENSG00000227124 | exonic (nonsynonymous SNV) | c.A293C: | p.Q98P | Uncertain significance | NA |
| chr3 | 75788226 | C/A | He | AR (compound heterozygote) | *ZNF717* | ENSG00000227124 | exonic (nonsynonymous SNV) | c.G548T: | p.C183F | Uncertain significance | NA |
| chr9 | 97522077 | G/T | He | AD (novel) | *C9orf3* | ENSG00000148120 | exonic (nonsynonymous SNV) | c.G12T: | p.Q4H | Uncertain significance | 9,36E-05 |
| chrX | 70330844 | G/A | Ho | X-linked | ***IL2RG*** | ENSG00000147168 | exonic (nonsynonymous SNV) | c.C172T: | p.P58S | Uncertain significance | NA |
| chrX | 71838564 | T/C | Ho | X-linked | *PHKA1* | ENSG00000067177 | exonic (nonsynonymous SNV) | c.A2188G: | p.M730V | Uncertain significance | 0,0011 |
| chrX | 82763621 | C/G | Ho | X-linked | *POU3F4* | ENSG00000196767 | exonic (nonsynonymous SNV) | c.C289G: | p.R97G | Uncertain significance | 0,0003 |

Abbreviations: Chr, chromosome; Ho, homozygous; He: heterozygous; NA, not available; AR: autosomal recessive; AD: autosomal dominant; kb, kilobases. The presented information has been retrieved using Annovar (accessed in May 2017). ^a^ Referring to ENSEMBL reference sequences (GRCh37.p13). ^b^ InterPro database. ^c^ Minor allele frequency according to gnomAD database. The frequency filtering was based on data from Genome Aggregation Database (gnomAD, Cambridge, MA, USA; http://gnomad.broadinstitute.org/; accessed in May 2017) and the national SISu project (http://sisu.fimm.fi/). In addition, we discarded all the variants exceeding the frequency of 0.05 according to an internal-use database. The rare variants affecting the coding regions were filtered based on the predicted consequences at the transcript level, with the selection of frameshift, nonsense, splicing and missense variants. All filtered variants were further evaluated according to the ACMG Standards and Guidelines and prioritized using the predicted effect on the protein, the conservation of the affected amino acids and in silico prediction tools. The data were analyzed with an in-house bioinformatics pipeline.

**Table S5** Germline variant in the *IL2RG* gene identified in a patient with recurrent respiratory infections and bronchiectasis

| **Nucleotide change^a^** | **Amino acid change^a^** | **Genotype** | **Inheritance** | **MAF^b^** | **Carriers (overall / Finland)^b^** | **REVEL score^c^** | **Classification^d^** | **Reference** |  |
| --- | --- | --- | --- | --- | --- | --- | --- | --- | --- |
| **c.172C>T** | **p.(Pro58Ser)** | **hemyzygous** | ***de novo*** | **-** | **-** | **0,699** | **Uncertain significance** | **novel** |  |

Abbreviations: MAF: minor allele frequency. Data retrieved with Annovar. ^a^ Location according to Ensembl (ENSG00000147168; ENST00000374202; GRCh37.p13);
^b^ Minor allele frequency according to gnomAD database; ^c^ REVEL pathogenicity score [3]; ^d^ estimated according to the ACMG Standards and Guidelines [4].

**Table S6** Surface expression IL2RG (CD132) on CD4+ and CD8+ lymphocytes, CD4+low monocytes, CD3-CD56- B cells and CD56+ NK cells measured by mean fluorescent intensity (MFI)

|  | **Control 1** | **Control 2** | **Patient** |
| --- | --- | --- | --- |
| **CD4+** | 2 199 | 2 471 | 981 |
| **CD8+** | 2 114 | 2 210 | 1 684 |
| **Monocytes** | 4 923 | 5 683 | 4 213 |
| **B cells (CD3-CD56-)** | 418 | 535 | 394 |
| **NK cells (CD56+)** | 1 109 | 1 595 | 781 |

**Table S8** Immunological characteristics of the second patient

|  | **Healthy control median/ reference range (cells/** **μl)** | **At age 14 days** | **At age 36 days** |
| --- | --- | --- | --- |
| **Leukocytes** | **4500-13500/ μl (HUSLAB)** | **7200/ μl** | **5700/ μl** |
| Lymphocytes | 3400-7600; 13-48% | 2981/ μl; 41.4% | 3363/ μl; 59% |
| Monocytes | 4-9% | 25.2% | 22% |
| Neutrophils | 36-77% | 25.7% | 15% |
| **CD3+ T cells** | **2500-5500/ μl; 53-84% (of lymphocytes)** | **1252/ μl; 42%** | **1379; 41%** |
| CD4+ T cells | 1600-4000/ μl; 35-64% | 596/ μl; 20% | 572/ μl; 17% |
| CD4+CD45RA+ | - | 49% | 71% |
| CD4+CD45RO+ | - | 38% | 15% |
| CD4+CD69+ | - | 1% | 0% |
| CD4+CD69- | - | 19% | 17% |
| CD8+ T cells | 560-1700/ μl; 12-28% | 626/ μl;21% | 807/ μl;24% |
| HLA-DR+CD4+ | - | 1% | 1% |
|  |  |  |  |
|  |  |  |  |
| HLA-DR+C8+ | - | 1% | 1% |
| HLA-ABC+ | - | 100% | 0 |
| TCRαβ^+^ CD4-CD8- | - | 0 | 0 |
| **CD4/8 ratio** | 0.8-3.7 | 0.95 | 0.71 |
| **CD19+ B cells** | **300-2000/ μl; 6-32% (of lymphocytes)** | **1103/ μl; 37%** | **1143/ μl; 34%** |
| CD19+CD5+ | - | 35% | 31% |
| CD19+CD27+ | - | 2% | 2% |
| CD20+ | - | 37% | 33% |
| CD20+CD27+ | - | 3% | 1% |
| **CD56+ NK cells** | **170-1100/ μl;4-18% (of lymphocytes)** | **447/ μl; 15%** | **504/ μl;15%** |
| CD56+CD3- | **-** | 15% | 15% |
| CD56+CD3+ | **-** | 0% | 0% |
| CD56+CD16+ | **-** | 11% | 12% |
| **Serum** IgG, IgA, IgM **levels** |  | Normal |  |
| T lymphocyte proliferative responses to mitogens and other stimuli |  | PHA decreased ConA normal  CD3 decreased,  CD3& IL-2 decreased |  |
| Specific antibodies against vaccine antigens |  | n/a |  |

**References**

[1]Trotta L, Hautala T, Hämäläinen S, Syrjänen J, Viskari H, Almusa H, et al. Enrichment of rare variants in population isolates: single AICDA mutation responsible for hyper-IgM syndrome type 2 in Finland. Eur J Hum Gen 2016;24:1473-1478

[2]Trotta L, Martelius T, Siitonen T, Hautala T, Hämäläinen S, Juntti H, et al. ADA2 deficiency: Clonal lymphoproliferation in a subset of patients. J Allergy Clin Immunol 2018;141:153-1537

[3]Sulonen A, Ellonen P, Almusa H, Lepistö M, Eldfors S, Hannula S, et al. Comparison of solution-based exome capture methods for next generation sequencing. Genome Biol 2011;12:R94

[4]Lek M, Karczewski KJ, Minikel EV, Samocha KE, Banks E, Fennell T, et al. Analysis of protein-coding genetic variation in 60,706 humans. Nature 2016;536:285-291

[5]Lim ET, Würtz P, Havulinna AS, Palta P, Tukiainen T, Rehnström K, et al. Distribution and medical impact of loss-of-function variants in the Finnish founder population. PLoS Gen 2014;10:e1004494

[6]Richards S, Aziz N, Bale S, Bick D, Das S, Gastier-Foster J, et al. Standards and guidelines for the interpretation of sequence variants: a joint consensus recommendation of the American College of Medical Genetics and Genomics and the Association for Molecular Pathology. Gene Med 2015;17:405-424
